# The role of ethanol oxidation during carboxydotrophic growth of *Clostridium autoethanogenum*

**DOI:** 10.1111/1751-7915.14338

**Published:** 2023-10-09

**Authors:** Martijn Diender, James C. Dykstra, Ivette Parera Olm, Servé W. M. Kengen, Alfons J. M. Stams, Diana Z. Sousa

**Affiliations:** ^1^ Laboratory of Microbiology Wageningen University & Research Wageningen The Netherlands; ^2^ Centre for Living Technologies Eindhoven‐Wageningen‐Utrecht Alliance Utrecht The Netherlands; ^3^ Centre of Biological Engineering University of Minho Braga Portugal

## Abstract

The Wood–Ljungdahl pathway is an ancient metabolic route used by acetogenic carboxydotrophs to convert CO into acetate, and some cases ethanol. When produced, ethanol is generally seen as an end product of acetogenic metabolism, but here we show that it acts as an important intermediate and co‐substrate during carboxydotrophic growth of *Clostridium autoethanogenum*. Depending on CO availability, *C. autoethanogenum* is able to rapidly switch between ethanol production and utilization, hereby optimizing its carboxydotrophic growth. The importance of the aldehyde ferredoxin:oxidoreductase (AOR) route for ethanol production in carboxydotrophic acetogens is known; however, the role of the bifunctional alcohol dehydrogenase AdhE (Ald–Adh) route in ethanol metabolism remains largely unclear. We show that the mutant strain *C. autoethanogenum* ∆*adhE1a*, lacking the Ald subunit of the main bifunctional aldehyde/alcohol dehydrogenase (AdhE, CAETHG_3747), has poor ethanol oxidation capabilities, with a negative impact on biomass yield. This indicates that the Adh–Ald route plays a major role in ethanol oxidation during carboxydotrophic growth, enabling subsequent energy conservation via substrate‐level phosphorylation using acetate kinase. Subsequent chemostat experiments with *C. autoethanogenum* show that the wild type, in contrast to ∆*adhE1a*, is more resilient to sudden changes in CO supply and utilizes ethanol as a temporary storage for reduction equivalents and energy during CO‐abundant conditions, reserving these ‘stored assets’ for more CO‐limited conditions. This shows that the direction of the ethanol metabolism is very dynamic during carboxydotrophic acetogenesis and opens new insights in the central metabolism of *C. autoethanogenum* and similar acetogens.

## Graphical abstract

Ethanol can be an end product of acetogenic carbon monoxide fermentation. Here we show that during carboxydotorphic growth of *C. autoethanogenum*, ethanol is not only an end product, but that is also acts as temporary storage for energy and reduction equivalents. When carbon monoxide availability becomes too low, the storage can be consumed, releasing the stored energy and reducing equivalents.

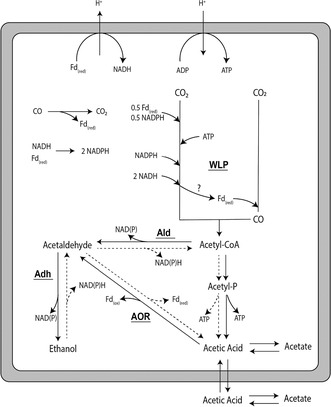

## INTRODUCTION

Microbial carbon monoxide metabolism (carboxydotrophy) has attracted attention over the last decade due to the interest in waste gas conversion to chemicals and fuels (Bengelsdorf et al., 2018; Köpke & Simpson, 2020; Liew, Martin, et al., 2016; Redl et al., 2017). Acetogenic bacteria play a role in such applications, enabling the production of alcohols and organic acids from CO‐rich gases. The Wood–Ljungdahl pathway (WLP) is central in carboxydotrophic acetogens and is used for both energy conservation and CO_2_ assimilation. Over the past years, the role of the Rnf complex (Müller et al., 2008; Tremblay et al., 2013) and several bifurcation mechanisms (Buckel & Thauer, 2013; Müller et al., 2018) in acetogenic metabolism have been uncovered. Moreover, acetogenic metabolism has been studied using metabolic models (Greene et al., 2019; Mahamkali et al., 2020; Valgepea et al., 2017, 2018) and knockout studies (Liew et al., 2017; Liew, Henstra, et al., 2016).

**FIGURE 1:**
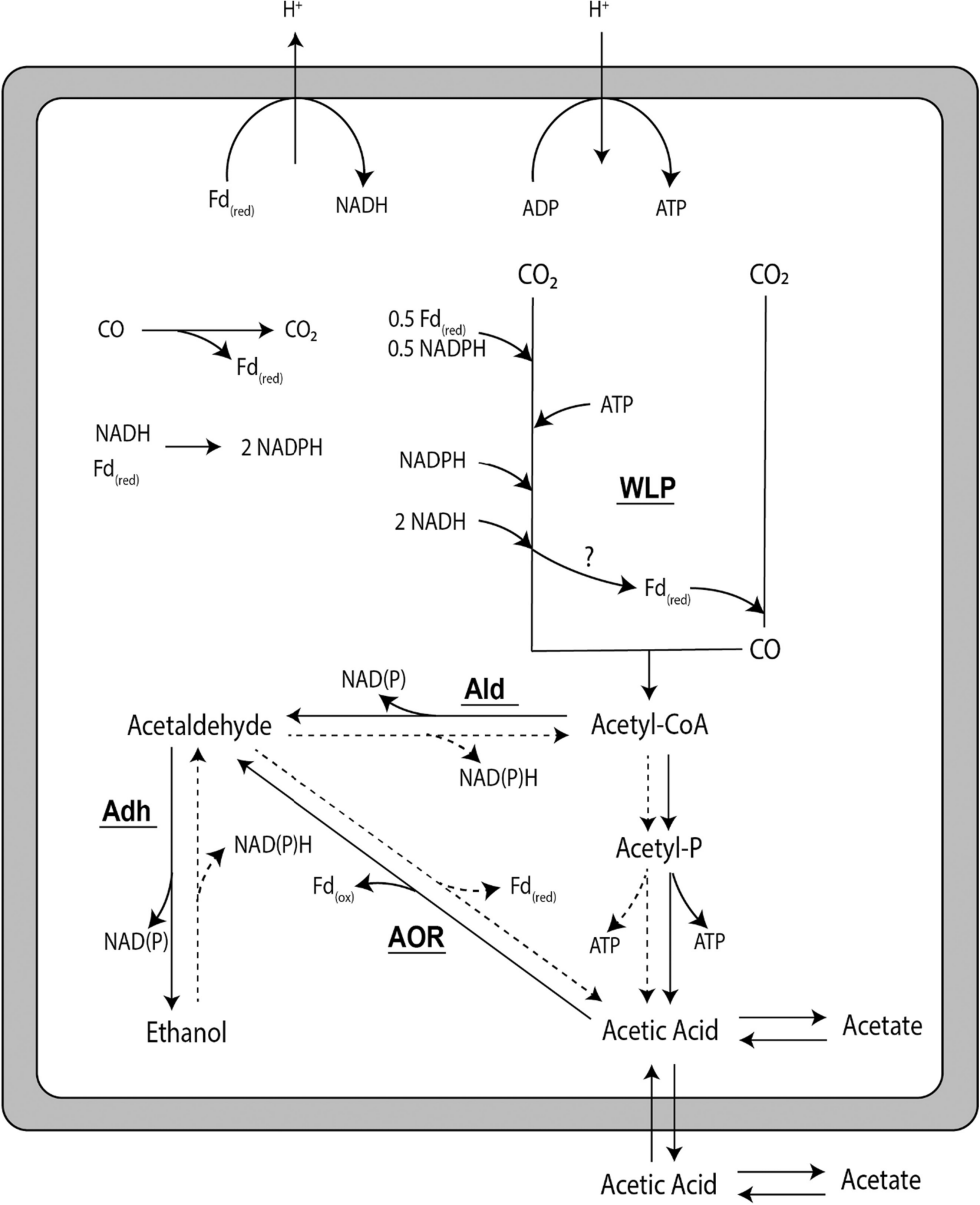
Depiction of carboxydotrophic central carbon metabolism in *C. autoethanogenum*. The overall metabolism is not displayed stoichiometrically. The pathways potentially used for ethanol oxidation (the AOR and/or Ald pathway) are shown as dashed lines. The bifurcation function of the methylene‐THF reductase is speculative and therefore indicated with a question mark (?). Adh, alcohol dehydrogenase; Ald, acetaldehyde dehydrogenase; AOR, aldehyde:ferredoxin oxidoreductase; WLP, Wood–Ljungdahl pathway.

In some carboxydotrophic acetogens, ethanol is considered a major metabolic end product that can be formed from acetyl‐CoA via two routes (Figure 1): (I) the aldehyde:ferredoxin oxidoreductase (AOR) route, using the phosphotransacetylase (Pta), acetate kinase (AckA) and subsequently AOR and alcohol dehydrogenase (Adh) to generate acetaldehyde and subsequently ethanol, or (II) the bifunctional AdhE (Ald–Adh) route, using Ald and Adh to produce acetaldehyde and subsequently ethanol (Köpke et al., 2010). Alcohol metabolism in carboxydotrophic acetogens such as *C. ljungdahlii* and *C. autoethanogenum* is strongly pH dependent (Abubackar et al., 2012; Abubackar, Bengelsdorf, et al., 2016; Safo et al., 2021) and is suggested to be regulated by thermodynamics rather than genetic or allosteric regulation (Diender et al., 2019; Mahamkali et al., 2020; Richter et al., 2016; Valgepea et al., 2017). Genetic knockout and modelling studies of *C. autoethanogenum* showed that the AOR pathway plays an important role in ethanol production during gas fermentation (Greene et al., 2019; Liew et al., 2017; Mahamkali et al., 2020; Valgepea et al., 2017). Via this route, starting from acetyl‐CoA, ATP is conserved via acetate kinase, after which reduced ferredoxin (Fd_(Red)_) and NAD(P)H are oxidized via AOR and Adh respectively. The role of the Ald–Adh pathway in ethanol production during gas fermentation is unclear, and disruption of the Ald subunit of AdhE1 even resulted in increased ethanol production (Liew et al., 2017). In some studies, the reoxidation of alcohols to their respective acids near the end of the exponential growth phase has been observed for cultures of acetogens utilizing CO (Diender et al., 2016; Köpke et al., 2010; Liew et al., 2017; Liu et al., 2020) or H_2_ (Mock et al., 2015). Additionally, it was shown that *A. woodii* could use ethanol as sole substrate by using its Ald–Adh pathway, mainly conserving energy via acetate kinase (Bertsch et al., 2016). This suggests that ethanol metabolism in gas‐fermenting acetogens is reversible and that ethanol potentially acts as a (co‐) substrate for acetogenesis during gas fermentation. While both Aor and Ald have been shown to play a role in ethanol metabolism (Liew et al., 2017), it remains unclear when alcohol (co‐)consumption occurs and how this impacts the growth characteristics of the microorganism.

We hypothesize that ethanol oxidation in AOR‐containing acetogens, such as *C. autoethanogenum*, mainly takes place via the Ald–Adh route under electron‐limiting conditions. This would be in line with the theory that ethanol production is a form of overflow metabolism (Allaart et al., 2023), and would allow for the reuse of reduction equivalents and energy stored during substrate‐abundant conditions and overall enables more efficient use of CO as a substrate.

The results presented here show that ethanol production and oxidation during carboxydotrophic growth of *C. autoethanogenum* are strongly affected by relative CO availability. Knockout of the Ald subunit of AdhE1 (∆a*dhE1a*) showed that this subunit plays an important role in ethanol oxidation to acetate. In addition, the results show a significantly higher biomass yield during co‐consumption of CO and ethanol in the wt versus Δ*adhE1a* strain, pointing towards a hitherto undescribed link between this pathway and energy conservation during carboxydotrophic growth of *C. autoethanogenum*. Additionally, the data indicate higher biomass yields under higher CO‐exposed conditions, suggesting the operation of a more efficient energy conservation route in *C. autoethanogenum* under these conditions. Together, the data suggest that *C. autoethanogenum* flexibly uses its acetogenic/solventogenic metabolism to optimize the energy yield and to balance its redox metabolism during carboxydotrophic growth and to cope with fluctuations in CO supply.

## EXPERIMENTAL PROCEDURES

### Strains and cultivation

*Clostridium autoethanogenum* JA1‐1 (DSM 10061) was purchased from the German Collection of Microorganisms and Cell Cultures (DSMZ). *C. autoethanogenum* ΔadhE1a was obtained from earlier work (Dykstra et al., 2022).

Unless mentioned otherwise, experiments were conducted in medium containing (per litre of medium) 0.9 g NH_4_CL, 0.9 g NaCl, 0.2 g MgSO_4_*7H_2_O, 0.75 g KH_2_PO_4_, 1.94 g K_2_HPO_4_*3H_2_O, 0.02 g CaCl_2_ and 0.5 mg resazurin. The medium was supplemented with the following trace elements (per litre of medium): 1.5 mg FeCl_2_*4H_2_O, 0.025 mg FeCl_3_*6H_2_O, 0.070 mg ZnCl_2_, 0.1 mg MnCl*4H_2_O, 0.006 mg H_3_BO_3_, 0.190 mg CoCl_2_*6H_2_O, 0.002 mg CuCl_2_*2H_2_O, 0.024 mg NiCl_2_*6H_2_O, 0.056 mg Na_2_MoO_4_*2H_2_O, 0.0035 mg Na_2_SeO_3_ and 0.2 mg Na_2_WO_4_. The medium was boiled and cooled on ice under N_2_ flow, after which 0.75 g L‐cysteine was added per litre of medium as a reducing agent. Unless stated otherwise, the pH was adjusted to 6 using NaOH and HCl. The medium was dispensed under N_2_ flow into glass serum bottles that were immediately sealed with rubber stoppers and aluminium caps. The headspace was filled with the desired gas (e.g. CO) to a final pressure of 150 kPa. The bottles were autoclaved immediately after preparation. Before inoculation, the medium was further supplemented with a vitamin solution at a 1:50 dilution containing (per litre) 1 mg biotin, 10 mg nicotinamide, 5 mg *p*‐aminobenzoic acid, 10 mg thiamin, 5 mg pantothenic acid, 25 mg pyridoxamine, 5 mg cyanocobalamine and 5 mg riboflavin. Other additives, such as yeast extract (0.5 g/L), ethanol and acetate, were added from sterile stock solutions. Unless stated otherwise, cultivation was performed at 37°C with shaking at 150 rpm.

Batch growth tests were conducted to assess strain maintenance and the consumption of ethanol and butanol. The experiments were performed in 121‐mL rubber‐stoppered serum bottles. Ethanol or butanol was added from sterile stock solutions to the intended final concentrations. Bottles were incubated upright with shaking at 150 rpm. The composition of the headspace was monitored over time using GC, while the liquid composition was analysed using HPLC.

A modified version of the medium was used to perform experiments for comparison of the Δ*adhE1a* strain and the wild‐type controls (Figures 3 and 4). This modified ATCC medium 1754 PETC (American Type Culture Collection, ATCC, Manassas, VA) contained (per litre) 1.0 g NH_4_Cl, 0.1 g KCl, 0.2 g MgSO_4_, 0.8 g NaCl, 0.1 g KH_2_PO_4_, 0.02 g CaCl_2_·2H_2_O, 0.25 g sodium acetate, 1 g yeast extract, 20 g 2‐(N‐morpholino)ethanesulphonic acid (MES), 0.5 mg resazurin, 0.75 g L‐cysteine·HCl, 10 mL trace element solution and 10 mL Wolfe's vitamin solution. The composition of the trace element solution (per litre) was 2 g nitrilotriacetic acid, 1 g MnSO_4_·H_2_O, 0.8 g Fe(SO_4_)_2_(NH_4_)_2_·6H_2_O, 0.2 g CoCl_2_·6H_2_O, 0.2 mg ZnSO_4_·7H_2_O, 0.02 g CuCl_2_·2H_2_O, 0.02 g NiCl_2_·6H_2_O, 0.02 g NaMoO_4_·2H_2_O, 0.02 g Na_2_SeO_4_ and 0.02 g Na_2_WO_4_·2H_2_O. The vitamin solution composition (per litre) was 2 mg biotin, 2 mg folic acid, 10 mg pyridoxine hydrochloride, 5 mg thiamine · HCl, 5 mg riboflavin, 5 mg nicotinic acid, 5 mg calcium pantothenate, 0.1 mg vitamin B12, 5 mg *p*‐aminobenzoic acid and 5 mg thioctic acid.

Experiments comparing the ethanol oxidation ability of the wild type and Δ*adhE1a* were performed in 250 mL rubber‐stoppered bottles containing 100 mL of medium. The 20 mM ethanol at the start was added from sterile stock solution after autoclaving. Bottles were incubated without shaking and gas and liquid composition tracked using GC and HPLC.

### Active cell tests

The metabolic shift between acid and alcohol production was studied in 16‐mL screwcap Hungate tubes. The tubes were made anoxic with N_2_, filled with 15 mL of active culture from a *C. autoethanogenum* bioreactor operated in steady state (see below) and then centrifuged for 20 min at 3000 g. The supernatant was replaced by 5 mL fresh medium (pH 5.8) free of yeast extract and vitamins. Acetate and ethanol were added at concentrations ranging from 0 to 50 mM (water was added in the case of 0 mM). CO and CO_2_ were introduced into the headspace to a desired final proportion ranging from 0% to 40% of each gas. The tubes were incubated horizontally in a shaker at an agitation speed of 60 rpm and at 37°C. Sampling was performed every 1 or every 2 h until depletion of CO (determined via GC). At the end of the experiment, the final pH was measured. All sets of experiments were performed in triplicate.

### Bioreactor operation

Cultivation of *C. autoethanogenum* in chemostats was performed in a 1.5‐L bioreactor (Applikon) with a liquid volume set to 750 mL. Comparative experiments of ∆*adhE1a* strain and the wild type were performed in 750‐mL bioreactors (HEL‐group) filled with 500 mL of liquid. Both systems were equipped with redox, as well as pH probes. The pH was controlled using 3 M KOH. Gas outflow rates were determined using a bubble counter. After sterilization, the reactors were connected to the control tower to regulate temperature (37°C) and pH. The reactors were flushed for 3 h with N_2_ at a rate of 20 mL/min to create anaerobic conditions. Immediately before inoculation, the N_2_ flow was changed to a CO/N_2_ flow. Additionally, vitamins, yeast extract and L‐cysteine were introduced into the reactor at the same concentrations as described for bottle cultivation. After reduction of the medium to below −300 mV, the reactor was inoculated with the culture in a 1:20 ratio. For continuous operation, a peristaltic pump (Masterflex) was used, applying a dilution rate of 0.028 h^−1^. The medium tank contained medium that was acidified using 30 mL 37% (w/w) HCl per 10 L of medium to prevent contamination. The medium vessel was continuously sparged with N_2_ (5 L/h) to ensure anaerobic conditions of the inflow medium. All the gas supply volumes and production rates mentioned throughout the text have been recalculated to correspond to 1 atm pressure and a temperature of 298 K.

### Analytical techniques

The liquid‐phase composition was analysed via high‐pressure liquid chromatography with a MetaCarb 67H column (Agilent Technologies). The column was operated at a temperature of 45°C with a flow rate of 0.9 mL/min. Detection was performed via a refractive index (RI) and UV detector. The eluent was 0.01 N H_2_SO_4_. In all cases, samples of 0.5 mL were taken and immediately centrifuged at 13,000 *g*. Subsequently, 0.4 mL supernatant was added to 0.6 mL 10 mM DMSO in 0.1 N H_2_SO_4_ solution. Concentrations below 0.1 mM could not be accurately quantified and are further referred to as trace amounts. For samples with low concentrations of alcohols (<1 mM), samples were analysed on a GC‐2010 (Shimadzu). The column (DB Wax UI, 30 m, 0.53 μM diameter) was operated at a temperature of 40°C for 5 min, with subsequent ramping to 200°C over 5 min and remaining at the higher temperature for 5 min. A flame ionization detector was used. Acetaldehyde concentrations in the liquid phase were determined using gas chromatography–mass spectrometry (GC–MS). GC–MS was performed using a Trace GC Ultra coupled to a DSQ mass spectrometer equipped with a quadrupole mass filter (Shimadzu). The GC was equipped with an RT Q‐bond column. The initial oven temperature was 50°C; after 2 min, the temperature was ramped to 200°C over 5 min and kept at this level for 5 min. Liquid samples were stored in airtight rubber‐stoppered vials of 1 mL and allowed to equilibrate with the gas phase for 1 h. Gas samples of 1 mL for injection into the GC–MS instrument were taken using a 1‐mL syringe.

For gas analysis of CO, H_2_ and CO_2_, gas samples of 0.2 mL were taken with a 1‐mL syringe and analysed in a Compact GC 4.0 (Global Analyser Solutions). CO and H_2_ were measured using a Molsieve 5A column operated at 100°C coupled to a Carboxen 1010 precolumn. CO_2_ was measured using an Rt‐Q‐BOND column operated at 80°C. Detection was performed in all cases via a thermal conductivity detector. To determine CO concentrations in the liquid phase, samples of 5 mL liquid broth were taken and injected into a gas‐tight, rubber‐stoppered glass vial of 7 mL. Samples were immediately stored at 4°C and left overnight to equilibrate the gas and liquid phases. Oxygen present in the vial combined with the low temperature caused inactivation of the microbes, preventing CO consumption. The CO present in the headspace of the vials was determined using GC‐PDD. The total CO present in the vials was recalculated from the headspace value using Henry's law. While this method cannot fully exclude CO consumption during the ‘storage’ phase, ‘in vivo’ values might deviate from the measured values, which is why ‘relative’ dissolved CO levels are shown in the figures, showing relative fold change compared to the average concentration during steady‐state operation.

The dry weight was determined by centrifuging a predetermined volume of culture broth (at least 10 mL) and washing the pellet in ultrapure water twice. Cells were then dried at 120°C in pre‐weighed aluminium baskets before reweighing.

## RESULTS

### *C. autoethanogenum* is capable of alcohol oxidation in the presence of CO

**FIGURE 2:**
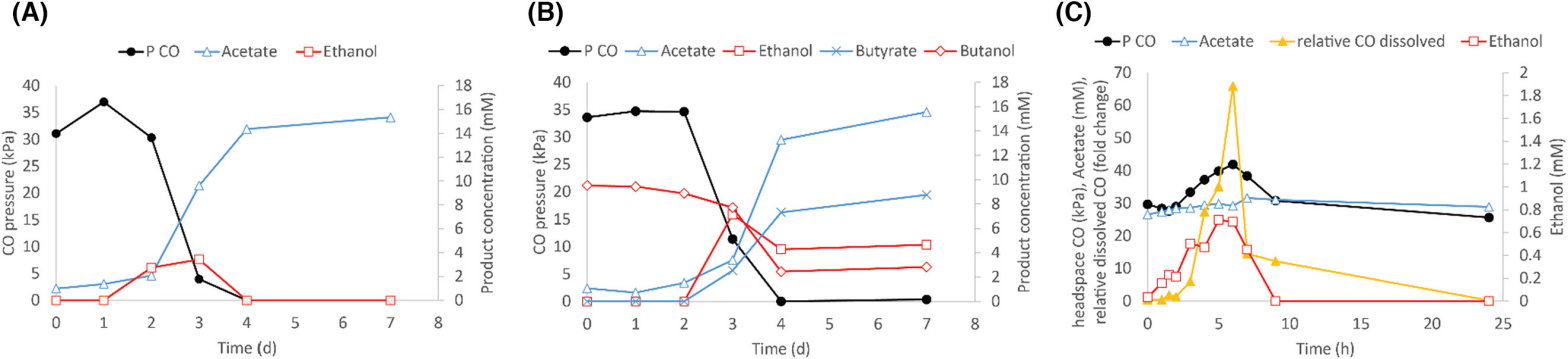
Alcohol production and subsequent oxidation by *C. autoethanogenum* during growth on CO. (A) Production and consumption of ethanol during carboxydotrophic growth of *C. autoethanogenum* in closed bottle tests. (B) Oxidation of butanol by *C. autoethanogenum* during carboxydotrophic growth in closed bottle tests. (C) Alcohol production and consumption by *C. autoethanogenum* in a chemostat during a CO spike (lasting from *t* = 0–6 h). Relative CO dissolved is shown in fold change relative to the average measured dissolved CO concentration during the initial steady state.

Carboxydotrophic growth of *C. autoethanogenum* resulted in the formation of ethanol and acetate (Figure 2A). However, when the CO pressure dropped (near the end of exponential growth), ethanol was oxidized back to acetate (Figure 2A). Similarly, butanol oxidation to butyrate was observed in CO‐grown cultures of *C. autoethanogenum* (Figure 2B). Alcohol oxidation did not take place further when CO was depleted (Figure 2B). It was further discovered that ethanol oxidation by *C. autoethanogenum* can occur to certain extend without CO, but only when CO_2_ is added as electron acceptor (Figure S1). Ethanol oxidation in this case halts before it can be fully depleted.

To study the effect of the CO/CO_2_ redox potential and availability of acetate on the switch between ethanol formation and oxidation, concentrated cell suspensions of *C. autoethanogenum* were exposed to different CO/CO_2_ ratios and acetate concentrations. Higher molar ratios of CO/CO_2_ (above 1.3) led to the formation of ethanol, while at lower CO/CO_2_ ratios (<1.3), oxidation of ethanol was observed (Figure S2). A clear effect of acetate concentration (ranging from 0 to 50 mM) could not be observed in this case.

To further test the effect of CO availability on ethanol production/consumption, growth experiments were performed in chemostats operated at pH 6 with a CO inflow rate of 1 mL/min. During steady operation over 4 days, acetate was the main product (25 ± 1.5 mM), ethanol was formed in trace amounts (<0.2 mM). A sudden increase in the CO inflow from 1 to 3 mL/min for 6 hours caused a peak in dissolved CO, reaching up to almost 70 times the steady‐state concentration (Figure 2C). Minutes after the initiation of the CO spike, traces of acetaldehyde (~100 μM) were detected in the medium (Figure S3), rapidly followed by ethanol formation up to 1 mM (Figure 2C). When the CO inflow rate was reverted to 1 mL/min after 6 h, the dissolved CO concentration dropped, and ethanol was rapidly oxidized back to acetate (Figure 2C).

### The acetaldehyde dehydrogenase pathway plays a major role in carboxydotrophic ethanol oxidation

The genome of *C. autoethanogenum* contains two AdhE‐encoding genes, AdhE1 (CAETHG_3747) and AdhE2 (CAETHG_3748), of which AdhE1 is thought to be the main AdhE, based on several fold higher transcript levels of AdhE1 compared to AdhE2 in autotrophic and heterotrophic growth conditions (de Lima et al., 2022; Diender et al., 2019; Marcellin et al., 2016; Mock et al., 2015; Valgepea et al., 2017). To examine the role of the Ald subunit of the bifunctional AdhE1 (CAETHG_3747) and study the pathway used for ethanol oxidation, a knockout mutant of the Ald subunit of the main AdhE was used (strain ∆*adhE1a*) (Dykstra et al., 2022).

**FIGURE 3:**
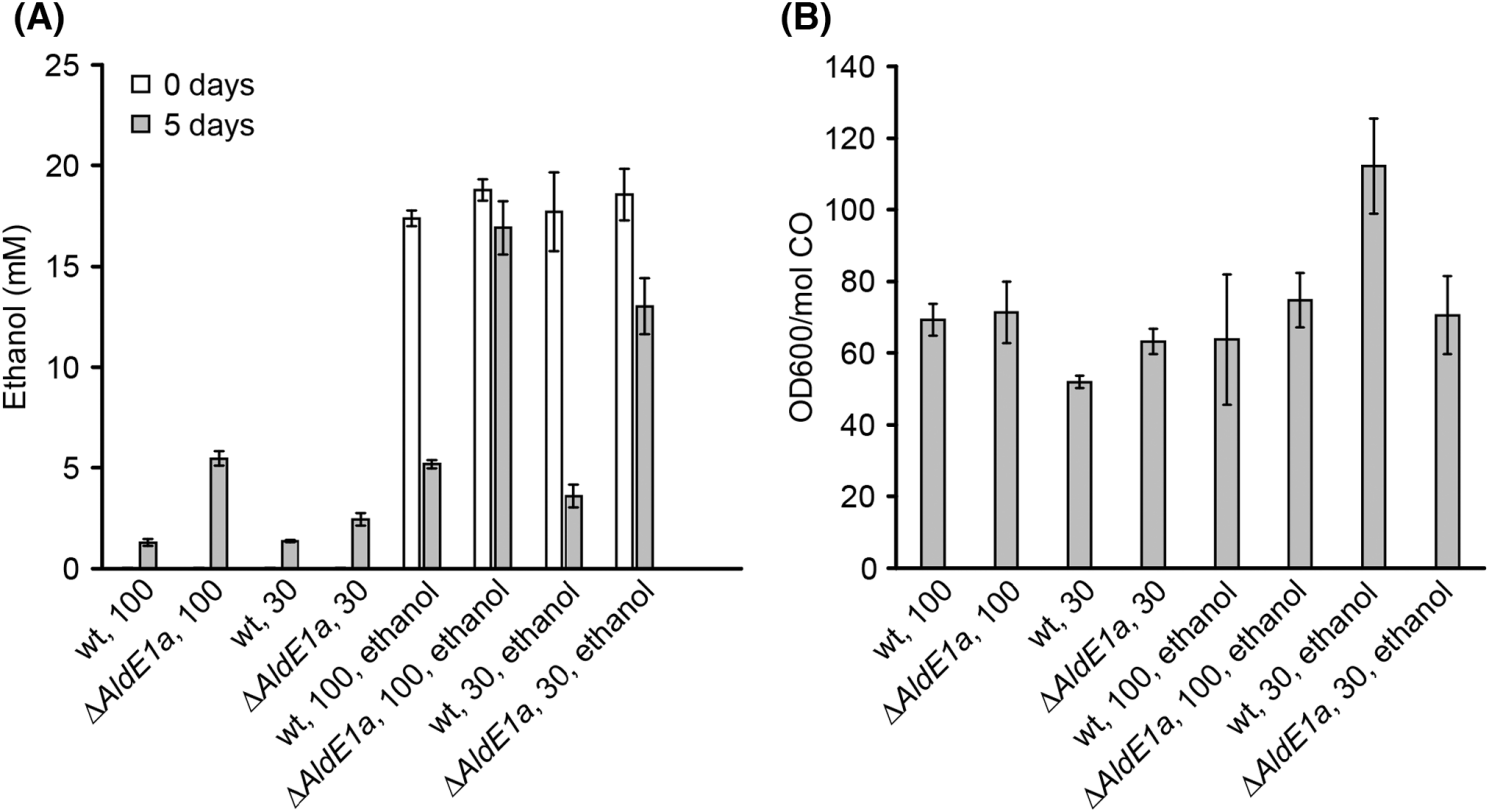
Comparison of the wild‐type (wt) and ∆adhE1a *C. autoethanogenum* strains in conditions with and without ethanol initially present and at 100 or 30 kPa CO pressure. Growth was finished after 5 days, and all CO present was consumed in all conditions. (A) Initial and final ethanol concentrations. (B) Final OD600 increase per mol CO consumed. Standard deviations are shown over triplicates.

The wild‐type and ∆*adhE1a* strains were both grown in batch at ‘high’ (100 kPa) or ‘low’ (30 kPa) CO partial pressure (pCO) in the presence (~20 mM) or absence of ethanol (Figure 3). Both the wild type and ∆*adhE1a* depleted the supplied CO completely within 5 days. When ethanol was not added, both the wild‐type and ∆*adhE1a* strain produced ethanol, both at high and low initial pCO, but production was significantly higher in the ∆*adhE1a* strain (Figure 3A). In the presence of ~20 mM initial ethanol, the wild‐type strain consumed 12–15 mM ethanol, while the ∆*adhE1a* strain consumed 2–5 mM ethanol, irrespective of the initial pCO (Figure 3A). Furthermore, the OD600 increase per CO consumed was similar among all conditions, except for the wild‐type strain at 30 kPa pCO with ethanol. In that case, the OD600 increase per CO consumed was almost twofold higher than that in incubations without ethanol supplementation (Figure 3B).

**FIGURE 4:**
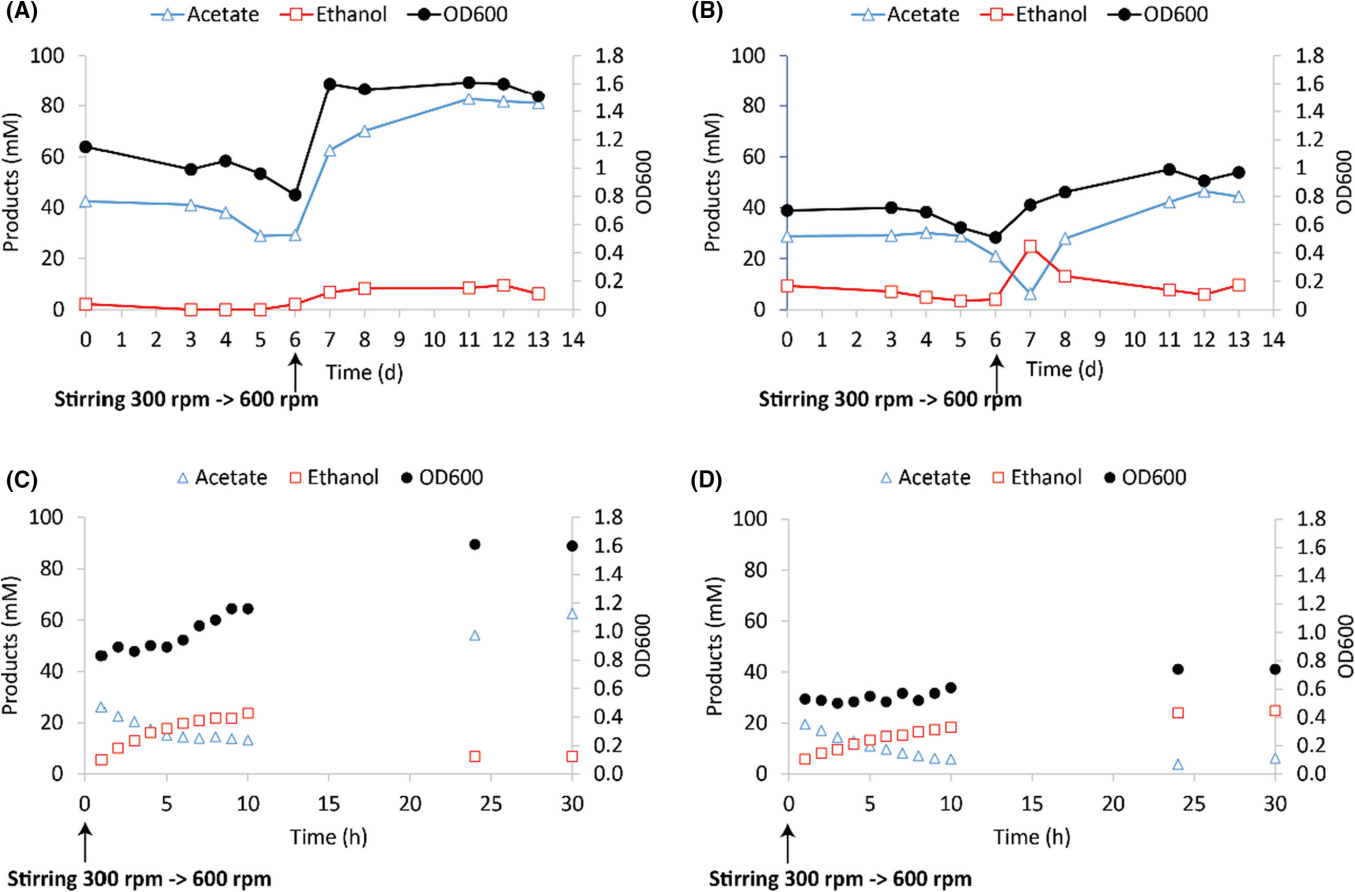
Production profile and OD600 measurements of the wild‐type and *∆adhE1a* *C. autoethanogenum* strains carboxydotrophically grown in a chemostat; stirring was ramped up from 300 to 600 rpm from day 2 onwards. (A) Wild type. (B) *∆adhE1a*. (C) Wild‐type first 30 h after stirring ramp. (D) *∆adhE1a* first 30 h after stirring ramp.

To further study the metabolic differences between the wild‐type and ∆*adhE1a* strain, both were grown carboxydotrophically in chemostats under identical conditions (pH 6, 1 mL/min CO inflow). Prior to exposing the cells to higher CO levels, the concentrations of acetate and ethanol for the wt were 34 ± 5.2 and 0.54 ± 1.2 mM respectively (Figure 4A). In contrast, strain ∆*adhE1a* produced 27 ± 4.2 mM acetate and 4.1 ± 1.9 mM ethanol (Figure 4B), evidencing a consistently higher background of ethanol production by the knockout strain. After increasing the stirring rate from 300 to 600 rpm, a higher CO transfer rate to the liquid was generated. As a result, ethanol production by the wild‐type and ∆*adhE1a* strain increased, largely due to the conversion of acetate to ethanol (Figure 4C,D). After 5 h, the wild type slowed ethanol production, after which OD600 increased rapidly (Figure 4C). Within 15 h, the wild‐type strain converted most of the generated ethanol back to acetate and significantly increased its OD600 from 0.9 ± 0.1 to 1.6 ± 0.05. Within the 24 h timeframe after the transfer rate increase, the ∆*adhE1a* strain did not convert ethanol back to acetate and showed a slower increase in OD600 (Figure 4D). In contrast to the 24 h response of the wt reactor, the chemostat with the ∆*adhE1a* strain required 2–3 days to remove the produced ethanol and reach a new OD600 of 0.96 ± 0.05 (Figure 4B). Based on the dilution rate of the reactor (0.67 d^−1^), ~51% and ~27% of the ethanol should be left in the system, after 1 and 2 days, respectively, if no consumption or production occurred. In the ∆*adhE1a* reactor, ethanol dropped from 24.8 to 13.2 mM after 1 day and to 7.7 mM after 2 days, a leftover of ~54% and 31%, respectively, indicating that no significant ethanol consumption occurred in this timeframe and the lowering ethanol concentration can be assigned to wash out rather than consumption. Several days after the stirring increase, the wt system showed a significant increase in OD, acetate and ethanol compared to the state before stirring rate change (*t*‐test, *p*‐value < 0.01), resulting in 82 ± 0.8 mM acetate and 8.1 ± 1.7 mM ethanol (Figure 4A). The ∆*adhE1a* also showed significantly differences in acetate concentration and OD after the stirring increase (*t*‐test, *p* < 0.01), resulting in concentrations of 44 ± 2.2 mM acetate and 7.8 ± 1.9 mM ethanol by the knockout strain (Figure 4B). This again shows ethanol preponderance in the knockout culture compared to the wt culture, potentially explained by the poor ethanol consumption ability of ∆*adhE1a*.

### Spikes in CO availability increase the biomass yield of *C. autoethanogenum*

**FIGURE 5:**
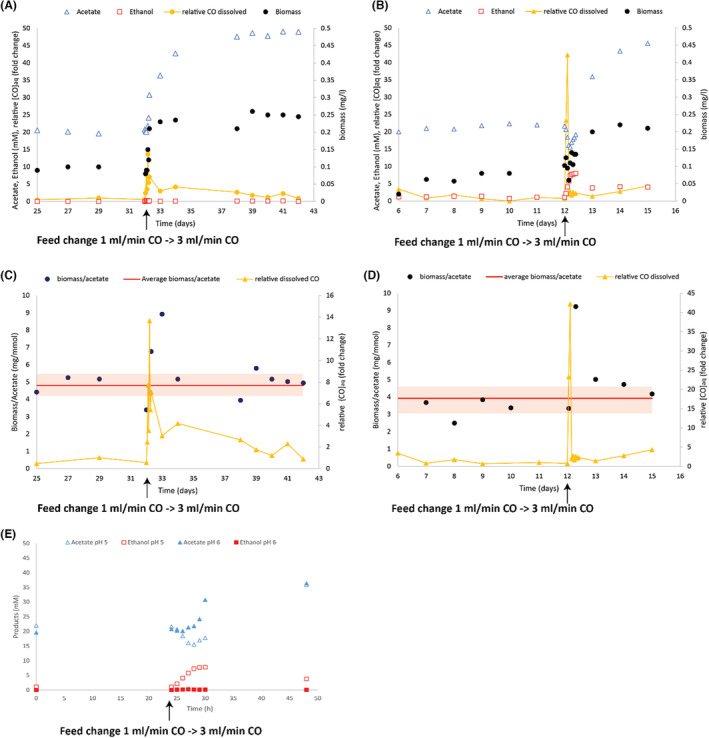
Production profile of wild‐type *C. autoethanogenum* grown in a chemostat reactor during an abrupt CO feeding rate increase from 1 to 3 mL/min. (A and C) Profile of the reactor operation at pH 6. (B and D) Profile of the reactor operation at pH 5. Average biomass/acetate levels (including standard deviation) are calculated over steady‐state conditions before and after the spike (and thus exclude the non‐steady‐state phase from day 32–34 and 12–14 respectively). The data shown are a snapshot of a longer chemostat run, hence the difference in time on the x‐axis. Relative CO dissolved are shown in fold change relative to the average measured dissolved CO concentration during the initial steady state. (E) The acetate and ethanol profile at pH 5 (open symbols) and pH 6 (closed symbols) around the moment of CO feed increase.

To study the effect of CO availability on the biomass yield of wild‐type *C. autoethanogenum*, additional chemostat experiments were performed. In steady state, with 1 mL/min CO inflow, 20 mM acetate was formed, traces of ethanol were detected and biomass accumulated to 0.10 ± 0.02 g dry weight/L. An increase in CO inflow to 3 mL/min caused a rapid increase in dissolved CO (Figure 5A). Similar to what was observed earlier (Figure 4), this resulted in ethanol production; however, levels still remained below 1 mM. This was followed by ethanol oxidation, a rapid acetate and biomass increase, and a drop in the dissolved CO concentration within 24 h (Figure 5A,E). After 3 days, new steady‐state values were reached (at 3 mL/min CO inflow), with 48 mM acetate, traces of ethanol and 0.24 ± 0.02 g/L dry weight biomass (Figure 5A). The ratio of biomass formed per acetate produced was ~5 mg biomass/mmol acetate in both the 1 and 3 mL/min steady‐state condition. Interestingly, the biomass concentrations reached steady‐state values much faster (within 6–24 h) than the acetate concentrations (~3 days). This caused a transient increase in biomass/acetate ratio to ~9 mg/mmol acetate (Figure 5C) before the value levelled off again at ~5 mg/mmol acetate. This suggests that during the short period of high CO availability, less acetate formation is required to sustain a similar production of biomass. During the first 24 h after the CO increase, ~0.1 g/L biomass was formed, and ~10 mM acetate was produced, whereas after reaching steady state, the generation of a similar amount of biomass required the production of ~20 mM acetate.

The same experiment was performed at a pH of 5. Here, before increasing the CO flow, 0.11 ± 0.03 g/L biomass, 22 mM acetate and traces of ethanol were found (Figure 5B). When CO was increased to 3 mL/min, the biomass concentration doubled overnight (to 0.2 ± 0.01 g/L dry weight). Immediately after the CO flow rate increase, instead of acetate formation as observed at pH 6, ethanol was formed from acetate present in the medium (Figure 5E). This caused a decrease in the acetate concentration to 15 mM, while ethanol increased to 8 mM (Figure 5B). Dissolved CO concentrations went down within 1 day to levels similar to the 1 mL/min condition. This contrasts with the pH 6 condition, which required several days to fully reduce the dissolved CO concentration. At pH 5, the biomass generated per acetate produced was only shortly altered after the spike and rapidly stabilized at a value similar to the previous steady‐state value (Figure 5D).

## DISCUSSION

For some acetogenic bacteria, ethanol is a major end product of carboxydotrophic acetogenic metabolism. Here, we report that *C. autoethanogenum*, depending on CO availability, can revert to ethanol consumption to perform acetogenesis (Figure 2). This finding is further supported by the observation that *C. autoethanogenum* can consume ethanol as a sole substrate (Figure S1), and that a lower CO/CO_2_ ratio leads to ethanol consumption (Figure S2). The specific switch point value observed here (CO/CO_2_ < 1.3) is likely dependent on environmental factors such as pH and ethanol/acetate concentrations. While acetate concentration appeared to have minimal impact on this variable, it is likely due to the limited range of acetate concentrations tested (0–50 mM). High CO availability, due to its strong reducing power, causes a more reduced ferredoxin pool that can be re‐oxidized by reducing acetate to ethanol. Low CO availability, and thus a likely more oxidized ferredoxin pool, results in the consumption of ethanol. Reducing equivalents derived from ethanol oxidation can subsequently be utilized in the metabolism (e.g. the WLP to reduce CO_2_ to acetate). The observation of simultaneous butanol oxidation (to butyrate) and reduction of acetate (to ethanol) during carboxydotrophic growth of *C. autoethanogenum* (Figure 2B) indicate that different acid/alcohol pairs can be oxidized and reduced simultaneously. The occurrence of these processes within the same bottle suggests that, in addition to the metabolic redox state, the reduction or oxidation of an acid/alcohol pair is also influenced by the concentration of the substrate and/or products. These observations, and the ability to rapidly alter the direction of alcohol metabolism upon changes in CO availability (Figure 2C), support the theory that acetogenic/solventogenic metabolism is strongly controlled by environmental parameters (e.g. redox pressure, pH, substrate and/or product concentrations; Abubackar et al., 2012; Abubackar, Fernández‐Naveira, et al., 2016; Mahamkali et al., 2020; Richter et al., 2016; Safo et al., 2021). Ethanol could be consumed as a sole substrate by *C. autoethanogenum* in the presence of CO_2_, yet the consumption of ethanol halts before reaching complete depletion (Figure S1). The inability to fully consume alcohol as sole substrate was also observed after carboxydotrophic growth (Figure 2B). This suggests that *C. autoethanogenum* possesses a limited capacity to use alcohols as sole substrate. However, the reasons for this observed behaviour remain uncertain. One possibility is that the accumulation of acidic products might render the conversion of the alcohol unfavourable. Ethanol consumption as sole substrate has previously been observed in *Acetobacterium woodii* (Bertsch et al., 2016). Given the distinct configurations of the WLP and cofactor utilization in *C. autoethanogenum* and *A. woodii*, it remains ambiguous whether these two microorganisms employ similar strategies for dealing with ethanol consumption.

Similar to ethanol production, ethanol oxidation can occur via two pathways in *C. autoethanogenum* (Figure 1; Figure S4): from ethanol to acetaldehyde and subsequently to acetate (using AOR) or from acetaldehyde via acetyl‐CoA to acetate (using Ald and Pta/AckA). The AOR pathway is expected to yield reduced ferredoxin and NADH, whereas the Ald pathway generates two NAD(P)H and one ATP via substrate‐level phosphorylation (Figure 1). Knockout of the Ald subunit of AdhE1 in *C. autoethanogenum* resulted in a hampered ability to consume ethanol (Figures 3 and 4). This suggests a key role for the Ald route in ethanol oxidation and aligns with earlier reports where disruption of the same Ald subunit of AdhE1 in *C. autoethanogenum* led to increased ethanol formation during batch growth on CO (Liew et al., 2017). Increased ethanol production, as also observed here (Figures 3 and 4), could be related to a decreased ethanol oxidation ability of the strain, resulting in higher net production of ethanol. Kinetic modelling studies of *C. autoethanogenum* predict that Ald operates towards the acetyl‐CoA direction under conditions where acetaldehyde is in excess (Greene et al., 2019). This might be the case in reductant‐abundant conditions (e.g. high dissolved CO concentrations) or conditions with relatively high ethanol concentrations. In line with these predictions, the data presented here indicate that Ald plays a role in the oxidation of ethanol to acetate. While limited, the ∆*adhE1a* strain can still convert ethanol back to acetate (Figure 3), indicating that in addition to the disrupted Ald subunit of AdhE1 (CAETHG_3747), other pathways contribute to ethanol oxidation. *C. autoethanogenum* carries genes for one additional AdhE with an Ald subunit (AdhE2, CAETHG_3748) and three additional monofunctional Ald genes (CAETHG_1819, 1830 & 3287), associated with gene clusters for bacterial microcompartments (BMCs), whose contribution to central carbon metabolism during growth on CO is not clear (Piatek et al., 2022). The AOR route likely also contributes to ethanol oxidation, as suggested for *Clostridium ljungdahlii* (Liu et al., 2020). Whereas AOR1 is proposed to be involved in ethanol production (Liew et al., 2017), AOR2 may have a role in alcohol oxidation (Liu et al., 2020), which agrees with increased ethanol production in the AOR2 knockout strain (Liew et al., 2017). However, near the end of fermentation, the AOR2 knockout strain still showed ethanol consumption, suggesting that AOR2 is not solely responsible for ethanol oxidation in *C. autoethanogenum*. Therefore, it is likely that in *C. autoethanogenum* as well as in *C. ljungdahlii*, alcohol oxidation can occur via both pathways depending on the environmental and metabolic conditions.

Cultures of *C. autoethanogenum* with less initial CO availability (30 kPa) showed an overall increased biomass yield per CO consumed during co‐consumption of ethanol (Figure 3). The ATP gain of *C. autoethanogenum* per CO consumed can be estimated for the co‐utilization of ethanol via the two different pathways (Figure S4). Assuming a bifurcating methylene‐THF reductase (Buckel & Thauer, 2018; Katsyv & Müller, 2020), the ATP yield per CO for acetogenesis is estimated as 0.38 ATP per CO (Figure S4A). Co‐consuming CO with ethanol in a 2:1 molar ratio using the Ald pathway results in a yield of 0.70 ATP per CO, an increase of ~1.88‐fold (Figure S4B). Using the reverse AOR pathway during the co‐consumption of 2:1 CO and ethanol results in 0.49 ATP per mol CO, an ~1.29‐fold increase (Figure S4C). The OD600/CO data (Figure 3) suggest that the wild‐type strain profits significantly from the co‐consumption of ethanol at lower CO availability, yielding ~1.5–2 times more biomass (OD600) per CO compared to other conditions. This is in line with the hypothesis that the Ald–Adh route has a major role in ethanol oxidation. The ∆*adhE1a* strain shows no significant OD600 change between solely CO‐based growth or co‐consumption of CO and ethanol (Figure 3), which can likely be explained by the poor ethanol consumption abilities of the ∆*adhE1a* strain. This shows that ethanol co‐consumption mainly promotes biomass yield at low CO availability, as a similar effect is not observed at a higher initial CO starting pressure (Figure 3). This is likely because the majority of reducing equivalents derived for energy metabolism come from CO under high‐CO initial conditions (>80%), potentially masking the contribution of ethanol‐derived energy conservation (<20%).

Solventogenic metabolism is strongly influenced by external parameters (such as redox pressure and pH), which can result in ‘forced’ production of ethanol under strong reducing conditions, ‘wasting’ electrons with a high energetic potential that could otherwise be invested in the WLP for energy conservation. While the direction of the Ald enzyme under alcohol‐producing conditions is still uncertain, results presented here indicate that under conditions with limited CO availability, the Ald enables reoxidation of ethanol, allowing access to reduction equivalents and energy stored. When exposed to increased CO availability during chemostat experiments, the ∆*adhE1a* strain responded similar to the wild type by producing ethanol (Figure 4). This is in line with the role of AOR in ethanol production. After some time (~5 h), the wild type slows ethanol production, followed by a rise in OD600, roughly doubling overnight (Figure 4). The ∆*adhE1a* strain is unable to re‐oxidize ethanol within the same timeframe and shows limited increase the OD600 (Figure 4). This shows that the ∆*adhE1a* strain is restricted in ethanol consumption and benefits less from reducing equivalents and energy stored in ethanol that it produces during excess reductant conditions. As a consequence, the ∆*adhE1a* strain allocates more reducing equivalents to ethanol and maintains a lower OD600 in chemostat reactors than the wild‐type strain (Figure 4). This suggests that the Ald route is also (partially) active in the acetaldehyde oxidizing direction under a steadier growth regime and could indicate that it is continuously used for balancing carboxydotrophic metabolism depending on the exact metabolic state of the cell.

Under high CO availability, *C. autoethanogenum* can maintain its redox balance by depositing electrons from ferredoxin to acetic acid via the AOR. The effect of increasing the CO supply on *C. autoethanogenum* at pH 6 compared to pH 5 reveals a more pronounced solventogenic behaviour at pH 5 (Figure 5). Interestingly, in the initial steady states, both systems show similar acetate concentrations. However, upon the introduction of additional CO, the relative higher levels of protonated acetic acid at pH 5 likely enable the cells to more rapidly transfer electrons to acetic acid via the AOR, resulting in increased ethanol formation (Figure 5E). In contrast, cells at pH 6 are exposed to less protonated acetic acid, and thus rely more on the rate of the WLP and the kinetics of acetic acid import. A way to potentially deal with this limitation is to immediately shuttle acetaldehyde formed by the AOR back to acetic acid via acetyl‐CoA using the Ald and Pta/Ack route. Such an ‘acetate cycle’, utilizing Aor, Ald and Pta/Ack, allows ferredoxin reoxidation, NAD(P)H production and ATP conservation via substrate‐level phosphorylation (Figure 1; Figure S4). When acetate, via acetaldehyde, is shuttled into the acetyl‐CoA pool, the ATP production would increase by approximately 1.8 times per acetate produced (Figure S4D). In chemostat experiments with the wild type at pH 6, we observed rapid formation of biomass when CO availability was suddenly raised (Figures 4 and 5). This resulted in a temporary increase in the biomass per acetate ratio (Figure 5C). A similar, but shorter, effect is seen at pH 5, potentially explained by the reoxidation of ‘stored’ ethanol when the dissolved CO concentrations drop (Figure 5D). The ∆*adhE1a* strain adapts more poorly to suddenly increased CO availability and is not able to ramp up its biomass production quickly after increased CO exposure (Figure 4). This suggests an important role of the Ald route in responding to quick changes in CO supply and potentially a role in promoting energy conservation. Earlier reports of the *C. autoethanogenum* ∆*adhE1a* strain also reported lower biomass formation compared to the wild type in general (Liew et al., 2017). Additionally, the data presented here indicate higher biomass yields under higher CO‐exposed conditions for the wt (Figure 5), suggesting the operation of a more efficient energy conservation route under these conditions. This is a potential novel mechanism of energy conservation in carboxydotrophic acetogens that requires further investigation.

As CO is a toxic substrate due to both its reductive and metal‐binding properties (Jeoung et al., 2014), the ability of acetogens to rapidly switch from acetogenesis to solventogenesis and back can be important for balancing redox and energy metabolism in changing environments. The constitutive expression of enzymes required for alcohol metabolism (Mahamkali et al., 2020; Richter et al., 2016; Valgepea et al., 2018) likely allows for such a fast response. Genetic control of this part of the metabolism might be too slow to react to sudden CO fluctuations. The data gathered here show the dynamic production and consumption of ethanol during carboxydotrophic growth are in line with the idea that solventogenesis is used as a form of overflow metabolism in these acetogens (Allaart et al., 2023). The Ald‐route appears to play a role in controlling the flux towards the acetyl‐CoA pool, ensuring that it remains replenished so that assimilatory metabolism and acetate production are not restricted during reductant‐abundant conditions. The solventogenic metabolism of *C. autoethanogenum* during carboxydotrophic growth thus appears to have three functions: (I) conversion of acids into their respective alcohols to ensure redox balance and limit the pH decrease; (II) oxidation of alcohols at low CO availability, allowing the release of stored reduction equivalents and energy; and (III) (potential) cycling of acetate via acetaldehyde/acetyl‐CoA to oxidize reduced ferredoxin, promote assimilatory metabolism and temporarily increase ATP yields under high‐CO (acetic acid limiting) conditions.

## AUTHOR CONTRIBUTIONS

**Martijn Diender:** Conceptualization (equal); formal analysis (lead); investigation (lead); methodology (equal); visualization (lead); writing – original draft (lead). **James C. Dykstra:** Investigation (equal); methodology (equal); writing – original draft (supporting). **Ivette Parera Olm:** Investigation (supporting); methodology (supporting); writing – review and editing (supporting). **Servé W. M. Kengen:** Supervision (equal); writing – review and editing (supporting). **Alfons J. M. Stams:** Conceptualization (equal); funding acquisition (equal); supervision (equal); writing – review and editing (equal). **Diana Z. Sousa:** Conceptualization (equal); funding acquisition (equal); supervision (lead); writing – review and editing (equal).

## FUNDING INFORMATION

Research presented in this article was funded by the Netherlands Science Foundation (NWO) (SynValue, project nr. ALWGK.2016.029), the NWO domain Applied and Engineering Sciences (AGS) (Perspectief Programma P16‐10), the Centre for living technologies (CLT) and the Netherlands Ministry of Education, Culture and Science (Project 024.002.002: Soehngen Institute of Anaerobic Microbiology).

## CONFLICT OF INTEREST STATEMENT

The authors declare that they have no conflict of interest.

## Supporting information


Data S1
