# Supplementary material for: The role of ethanol oxidation during carboxydotrophic growth of Clostridium autoethanogenum
Source: Microb Biotechnol. 2023 Oct 9;16(11):2082–93. doi: 10.1111/1751-7915.14338 (PMC10616641; doi:10.1111/1751-7915.14338)
Supplement: Supplementary file 1 — Data S1 [file MBT2-16-2082-s001.docx]

**Supplementary figures**

Figure S1.

Production profile of *C. autoethanogenum* growing solely on Ethanol as a substrate in presence of CO_2_. Standard deviation shown over duplicates

Figure S2

Validation of alcohol production and oxidation using active cells at different acetate concentrations and CO/CO_2_ ratios in the headspace. Black bordered markers are samples verified to be in alcohol oxidation stage, while borderless markers are verified to be in alcohol production stage. Different colors indicate each of eight different experimental conditions, with the different markers (triangles, squares or circles) of the same color being separate replicates of that condition. Samples of which the alcohol oxidation/production stage could not be verified, were not included in the figure (resulting in deviating number of sampling points across the different conditions).

Figure S3

Acetaldehyde formation in the first 15 minutes of CO increase in a culture of *C. autoethanogenum* in a chemostat run (figure 2C). Black dots indicate the acetaldehyde levels in the spike condition, grey dots indicate acetaldehyde levels of a 15m interval in a non-spike condition.

Figure S4

Estimations of ATP yield per CO and per acetate in different metabolic situations. Here it is assumed that the methylene-THF reductase is a bifurcating complex and that the Ald and Adh use NADH as cofactor.”

*
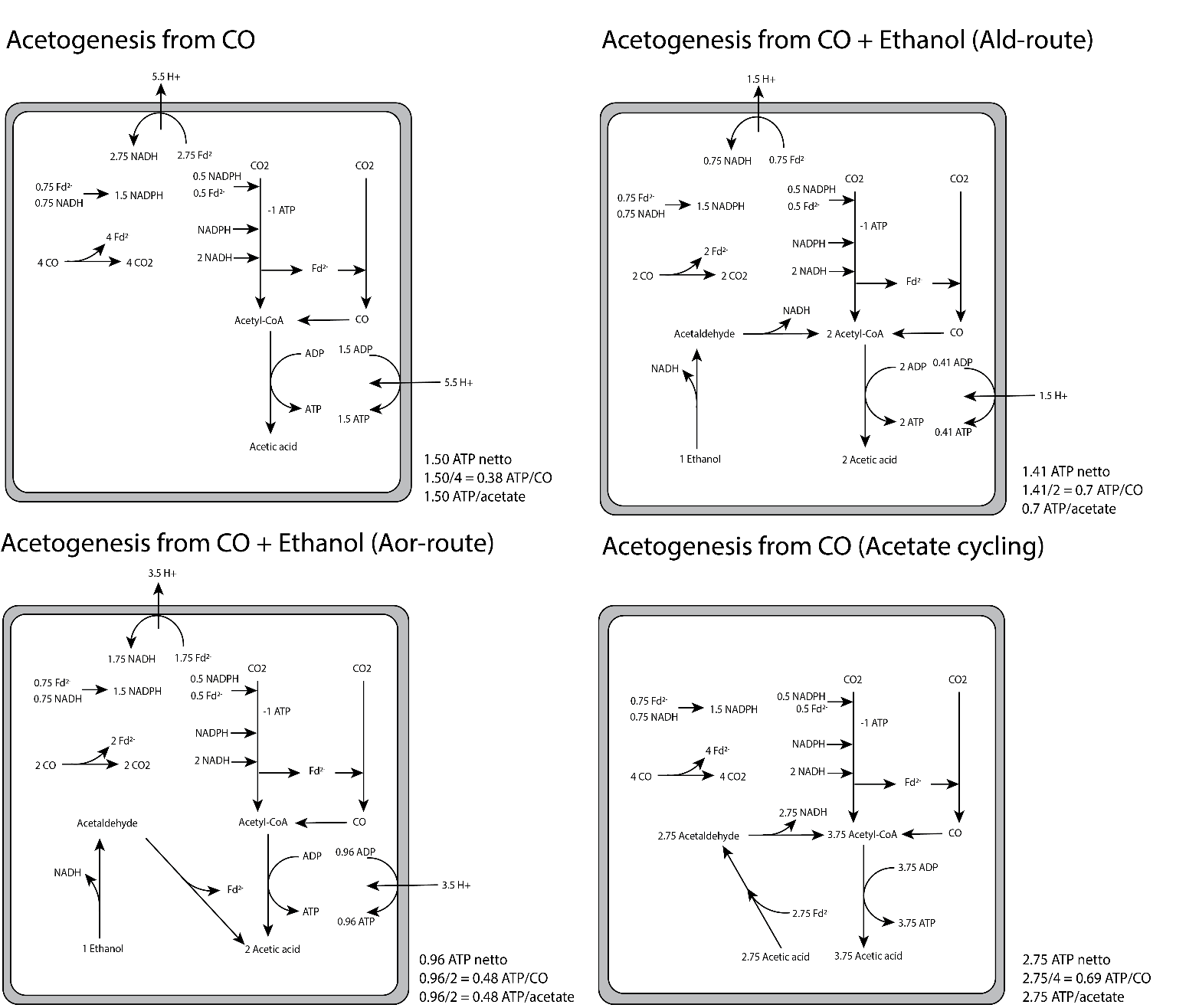
*
